# The impact of resveratrol and hydrogen peroxide on muscle cell plasticity shows a dose-dependent interaction

**DOI:** 10.1038/srep08093

**Published:** 2015-01-28

**Authors:** Alessandra Bosutti, Hans Degens

**Affiliations:** 1 School of Healthcare Science, Manchester Metropolitan University, Manchester, United Kingdom

## Abstract

While reactive oxygen species (ROS) play a role in muscle repair, excessive amounts of ROS for extended periods may lead to oxidative stress. Antioxidants, as resveratrol (RS), may reduce oxidative stress, restore mitochondrial function and promote myogenesis and hypertrophy. However, RS dose-effectiveness for muscle plasticity is unclear. Therefore, we investigated RS dose-response on C2C12 myoblast and myotube plasticity 1. in the presence and 2. absence of different degrees of oxidative stress. Low RS concentration (10 μM) stimulated myoblast cell cycle arrest, migration and sprouting, which were inhibited by higher doses (40–60 μM). RS did not increase oxidative capacity. In contrast, RS induced mitochondria loss, reduced cell viability and ROS production, and activated stress response pathways [Hsp70 and pSer36-p66(ShcA) proteins]. However, the deleterious effects of H_2_O_2_ (1000 µM) on cell migration were alleviated after preconditioning with 10 µM-RS. This dose also enhanced cell motility mediated by 100 µM-H_2_O_2_, while higher RS-doses augmented the H_2_O_2_-induced impaired myoblast regeneration and mitochondrial dehydrogenase activity. In conclusion, low resveratrol doses promoted *in vitro* muscle regeneration and attenuated the impact of ROS, while high doses augmented the reduced plasticity and metabolism induced by oxidative stress. Thus, the effects of resveratrol depend on its dose and degree of oxidative stress.

In response to damage, satellite cells rapidly undergo several cycles of cell division prior to withdrawal from the cell cycle to terminally differentiate and fuse with the damaged skeletal muscle fibres^1^. Also during hypertrophy, activation of satellite cells is considered to play a crucial role to maintain the myonuclear domain size by adding new myonuclei to the growing muscle fibres^2^. An adequate function of these cells thus appears essential for muscle maintenance, repair and growth^1^,^2^.

Effective regeneration and training adaptations critically depend on the level of generated reactive oxygen species (ROS)^3^,^4^. Mitochondria are considered the major site of ROS (i.e. superoxide anion) generation in tissues, but ROS are also derived from the enzymatic activation of cytochrome p450, NAD(P)H oxidase, xanthine oxidase and inflammatory activity^3^,^4^,^5^. In healthy skeletal muscle, xanthine oxidase, calcium-dependent and calcium-independent phospholipase (PL) A2 and putative NAD(P)H oxidase enzymes of the plasma membrane, triads and transverse tubules, are key players in superoxide generation in response to contractile activity^6^,^7^.

ROS can activate a number of signalling pathways that have an impact on cell migration, cell cycle transition, cell survival, apoptosis and differentiation^5^,^8^, all crucial for tissue repair. At low-to-moderate levels, ROS stimulates tissue healing and maintenance of muscle^4^, but if ROS generation persists for too long and at too high levels, it can delay tissue repair and even worsen the injury^9^,^10^,^11^,^12^. Such a situation of oxidative stress can develop from mitochondrial instability, an increase in oxidant exposure, and/or less effective endogenous antioxidant systems^7^,^13^.

To combat oxidative stress and its consequences, dietary supplementation with antioxidants has often been applied. Antioxidant supplementation has been shown to improve expression of anti-oxidant enzymes, muscle function and muscle repair^9^,^13^,^14^,^15^.

In this context, the anti-oxidant and anti-inflammatory polyphenol resveratrol (RS), commonly found in the skin of grapes and in other red fruits, has received extensive attention in the last years, showing cell-protection from oxidative stress-induced damage and inflammation with benefits in a variety of human diseases, including cancer, cardiovascular diseases and aging^14^,^15^,^16^,^17^,^18^. It has been shown that RS supplementation may convey resistance to oxidative stress by diminishing oxidative mitochondrial membrane damage and death of skeletal muscle cells^19^. Furthermore, it has been shown to prevent the catabolic effects of dexamethasone in myotubes^20^ and attenuate muscle atrophy in tumour-bearing mice^21^. Some studies even suggest that it can act as an exercise mimetic^22^. It has been reported, for instance, that RS prevented the decrease in muscle mass, function and oxidative capacity during muscle unloading^22^, and promoted *in vitro* myogenesis and hypertrophy, at least partly via regulation of expression of myogenic regulatory factors and cell cycle progression factors^23^.

Despite these reported benefits, many studies show weak beneficial effects and there is even an increasing awareness of detrimental side effects of RS^24^,^25^,^26^,^27^. In particular, RS has been shown to exert divergent effects on cell proliferation, differentiation and apoptosis, blunting or stimulating mitochondrial damage and ROS production^28^,^29^,^30^,^31^,^32^,^33^,^34^. These divergent effects are probably dependent on the cell type, organism, duration and dose of resveratrol exposure^33^ and/or the presence or absence of oxidative stress. Surprisingly, the literature contains little information about the effects of resveratrol on muscle cell plasticity in the presence or absence of oxidative stress.

The aim of the present study was to assess the effects of resveratrol on myoblast and myotube plasticity and to what extent these effects differ in the presence or absence of oxidative stress. Thereto, we explored in mouse skeletal muscle-derived C2C12 myoblasts and myotubes the dose response relationship of resveratrol on key phases of skeletal muscle remodelling and oxidative metabolism, in the presence or absence of hydrogen peroxide (H_2_O_2_), as a model of oxidative stress.

Our findings support the notion that low concentrations of ROS enhance myoblast cell migration while it is impaired at high concentrations. Similar to ROS, high doses of RS had detrimental effects on muscle cell viability, mitochondria stability and muscle plasticity, while low doses stimulated cell migration, cell cycle arrest and formation of cell sprouts.

## Results

### Effects of resveratrol

#### The effects of resveratrol on C2C12 myoblast remodelling

**Figure 1:**
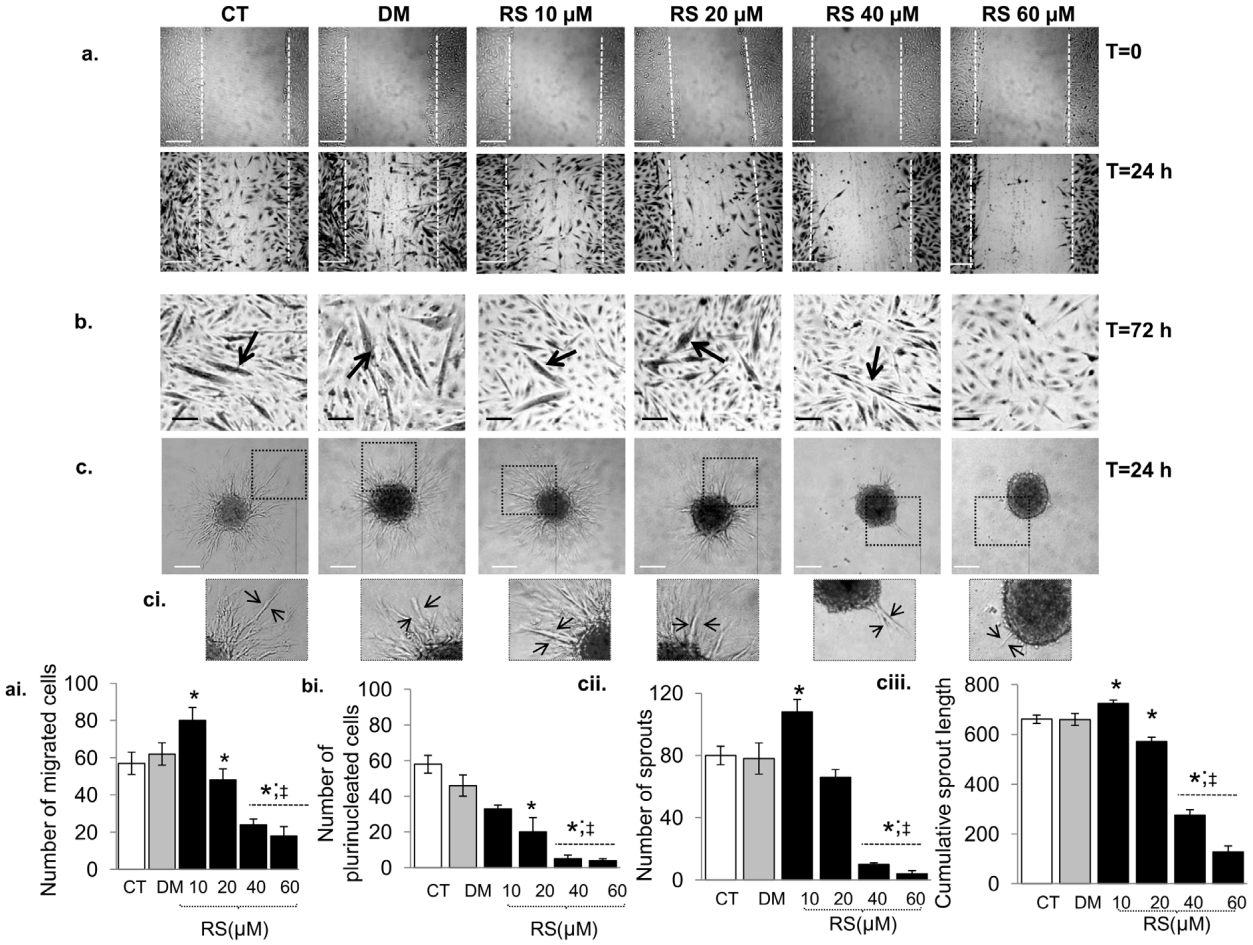
The effects of Resveratrol on C2C12 myoblast remodelling. Phase contrast images showing the impact of different concentrations of resveratrol (RS) on (a) C2C12 myoblast migration at t = 0 (basal) and at t = 24 h, (b) cell fusion to pluri-nucleated cells (myotubes) and (c and ci) sprout formation on spheroids. Figure ai, bi, cii and ciii show the number of migrated cells, pluri-nucleated cells, sprouts and cumulative sprout length, respectively. The effect of RS was dose-dependent. (a, ai) RS (10 µM) enhanced cell motility, while it was increasingly inhibited by increasing doses. A low dose of RS (10 µM) also enhanced sprout formation (cii, ciii), while high doses of RS (20–60 µM) markedly impaired the regenerative capacity as reflected by a lower number of pluri-nucleated cells (b and bi) and cell sprouts (cii) and sprout length (cii). Data are expressed as mean ± s.e.m. *: *P*<0.01 vs. CT; ‡: *P*<0.001 vs. RS 10 µM. DM vs. CT: in none of the cases significant. *P*-value calculated using a two-tailed Student's *t*-test. Bars 20 µm. Original magnification in ci, x 200. Each experiment was performed in triplicate.

We tested the impact of different concentrations of RS (10, 20, 40 and 60 μM) on cell proliferation, cell cycle progression (Supplementary Fig. 1), cell migration (Fig. 1a), cell fusion (Fig. 1b) and the quantity and quality of neo-formed sprouts (Fig. 1c, ci). The latter were characterized by the thickness, number (Fig. 1cii), and cumulative length (Fig. 1ciii) of sprouts originating from single spheroids. Changes in cell cycle progression were indicative of the potential of RS to modulate cell cycle arrest, an essential step in the early stage of differentiation (Supplementary Fig. 1).

We found that the effects of RS were dose-dependent. A low dose of RS (10 µM) enhanced cell motility, as seen by the increased number of migrated cells in scratched monolayers, while it was increasingly inhibited by increasing doses (Fig. 1ai). The degree of cell fusion was progressively inhibited by increasing concentrations, as revealed by the lower number of plurinucleated cells (myotubes; Fig. 1 bi). Ten µM RS stimulated the formation and quality of sprouts, as indicated by their increased number (Fig. 1cii), length (Fig. 1ciii) and thickness. However, with increasing doses, cell fusion was progressively impaired, and the number and length of cell sprouts progressively reduced (Fig. 1cii,ciii).

High concentrations of RS (40–60 µM) induced thinner and elongated myoblasts with an increased appearance of intracellular vesicles (data not shown) and reduced cell number (Supplementary Fig. 1a–c). Compared to controls, after 48 h only 10 µM RS enhanced the proportion of cells in G0+G1 phases and consequently reduced the proportion of cells in S+G2 phases (Supplementary Fig. 1d,e), suggesting cell cycle arrest^23^,^34^. Higher concentrations (40–60 µM) showed a decreased proportion of cells in the G0+G1 phases as early as after 24 h of treatment, with the significant appearance of a sub-G1 peak indicating apoptosis/necrosis (Supplementary Fig. 1d,e). Additionally, we found that RS-treated cells (20–60 µM) showed a concentration-dependent reduction of intracellular ROS (Supplementary Fig. 2) that was not significant at lower doses (10 µM).

Several *in vitro* studies have shown that RS can exert a cytotoxic effect, including induction of mitochondrial apoptosis *via* mitochondrial membrane depolarization^29^,^30^,^31^. Therefore, we analysed whether the observed cytotoxic effect of RS on C2C12 myoblasts were explicable by an effect of the compound on the stability of mitochondrial membrane potential (ΔΨm). In line with the increased apoptosis (Supplementary Fig. 1) 24 h treatment with RS induced mitochondrial membrane depolarisation (UR+LR panels; Supplementary Fig. 3ai), particularly at higher doses (40 and 60 µM), and this was associated with a significant reduction of cell viability (Supplementary Fig. 3b).

#### Effects of resveratrol on metabolic state and succinate dehydrogenase activity

**Figure 2:**
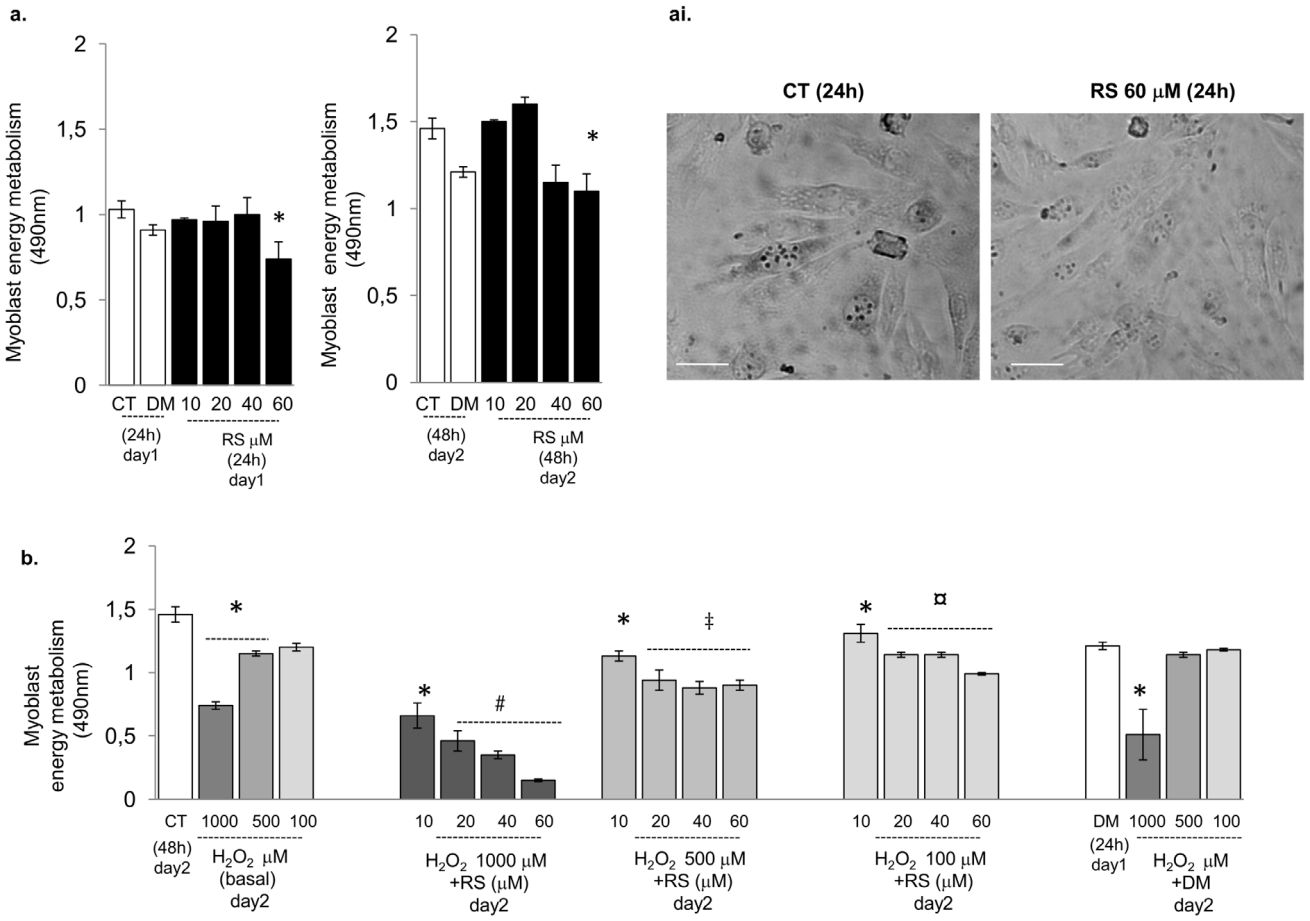
Resveratrol did not improve myoblast metabolic state and further enhanced the inhibitory effect of H_2_O_2_. (a) Effects of 24 and 48 h of resveratrol treatment on C2C12 myoblast energy metabolism. Cells were cultured 24 h and 48 h in the absence (CT) or presence of RS (10, 20, 40 and 60 µM), or DM vehicle. (ai) Phase contrast images showing a representative cellular formazan staining in CT and cell treated with 60 µM RS for 24 h. (b) In the experiment of preconditioning, 24 h treatment with scalar amount of resveratrol (day1) was followed by 24 h treatment with 100, 500 or 1000 µM H_2_O_2_ (day2). Treatment with H_2_O_2_ alone was also performed at day2. (b) Data from H_2_O_2_ alone and RS preconditioning were compared to CT at day2. (a,b) Irrespective of the period of incubation, 10 and 20 µM RS did not affect myoblast energy metabolism, while it was significantly reduced by 60 µM RS. Only 1000 and 500 µM H_2_O_2_ caused a reduction in energy metabolism, which was further hampered in a dose-dependent manner by RS-preconditioning. (b) Data are expressed as mean ± s.e.m. of biological triplicate. *P*-values calculated using a two-tailed Student's *t*-test. *: *P*<0.01 vs CT; #: *P*<0.001 vs H_2_O_2_ 1000 µM; ‡: *P*<0.01 vs H_2_O_2_ 500 µM; ¤: *P*<0.01 vs H_2_O_2_ 100 µM. Original magnification, x 50. Bars 20 µm.

**Figure 3:**
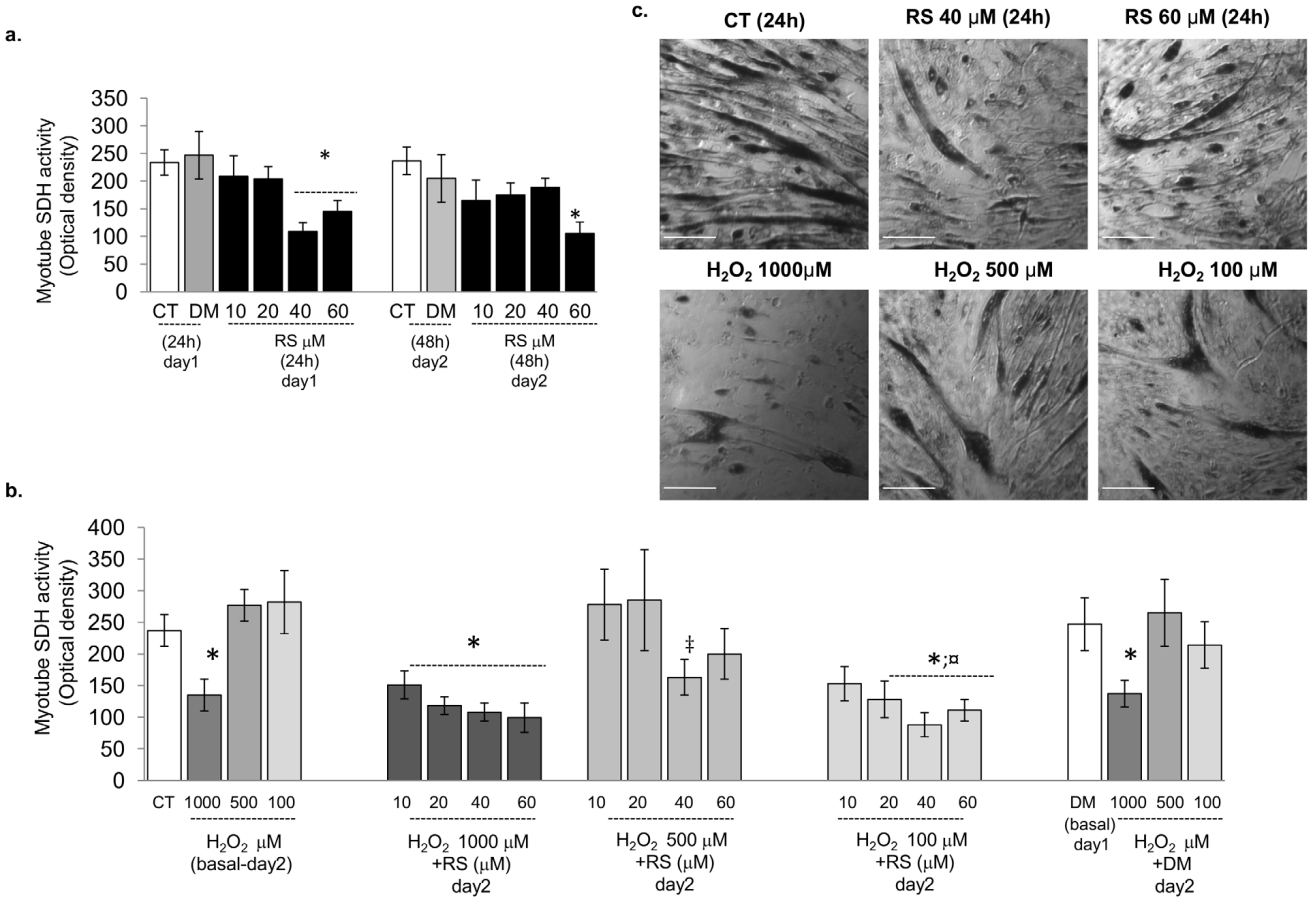
Resveratrol did not improve myotube oxidative capacity. (a) Effects of 24 and 48 h of resveratrol treatment on C2C12 myotube succinate dehydrogenase (SDH) activity. Myotubes at the 8^th^ day of differentiation were cultured 24 or 48 h in the absence or presence of RS (10, 20, 40, 60 µM) or vehicle (DM). (b) For the experiment of RS preconditioning, 24 h of treatment with scalar amount of resveratrol (day1) was followed by 24 h of treatment with 100, 500 or 1000 µM H_2_O_2_ (day2). Treatment with H_2_O_2_ alone was also performed at day2. In the graphs: day1 and day2 corresponding to the 9^th^ and the 10^th^ day of differentiation. Data from H_2_O_2_ alone and RS preconditioning experiment were compared to controls (CT) analysed at day2. (c) Representative phase contrast images showing SDH staining in CT and RS 40–60 µM or 1000, 500 and 100 µM H_2_O_2_-treated cells (24 h). (a) Ten and 20 µM RS alone did not significantly affect myotube SDH activity, but at higher doses (40 or 60 µM) it caused a reduced SDH activity. (b) 1000 µM H_2_O_2_ did reduce SDH activity, which was further aggravated by pre-treatment with increasing doses of RS. Data are mean ± s.e.m. of biological triplicate. *P*-values calculated using a two-tailed Student's *t*-test. *: *P*<0.01 vs CT;.‡: *P*<0.01 vs H_2_O_2_ 500 µM; ¤: *P*<0.01 vs H_2_O_2_ 100 µM. DM vs. CT in none of the cases significant. Original magnification x50. Bars 20 µm.

Mitochondrial depolarization may lead to a reduction in the number of mitochondria. Succinate dehydrogenase (SDH) activity has a fundamental function in oxidative energy metabolism^35^ and may be indicative of mitochondrial content and cell viability^36^. We examined the effect of RS on the active metabolic state in myoblasts (Fig. 2) and oxidative capacity in myocytes (SDH activity; Fig. 3). The active metabolic state was examined in cell myoblasts and not in myotubes, since the method required the accurate seeding of equal cell density for each condition, which is not possible for fused myocytes after 8 days of differentiation. C2C12 myoblasts (for active metabolic state; Fig. 2a) and myotubes (for SDH activity; Fig. 3a) were cultured 24 h and 48 h in the absence or presence of RS scalar amount. Figure 2a, shows that, irrespective of the period of incubation, 10 and 20 µM RS did not significantly affect energy metabolism, but higher doses (40 or 60 µM) caused a reduction in metabolic state in myoblasts (60 µM; Fig. 2a) and oxidative capacity in myotubes (40 or 60 µM; Fig. 3a).

#### Resveratrol modulates myosin type1 and total myosin ATPase activity

**Figure 4:**
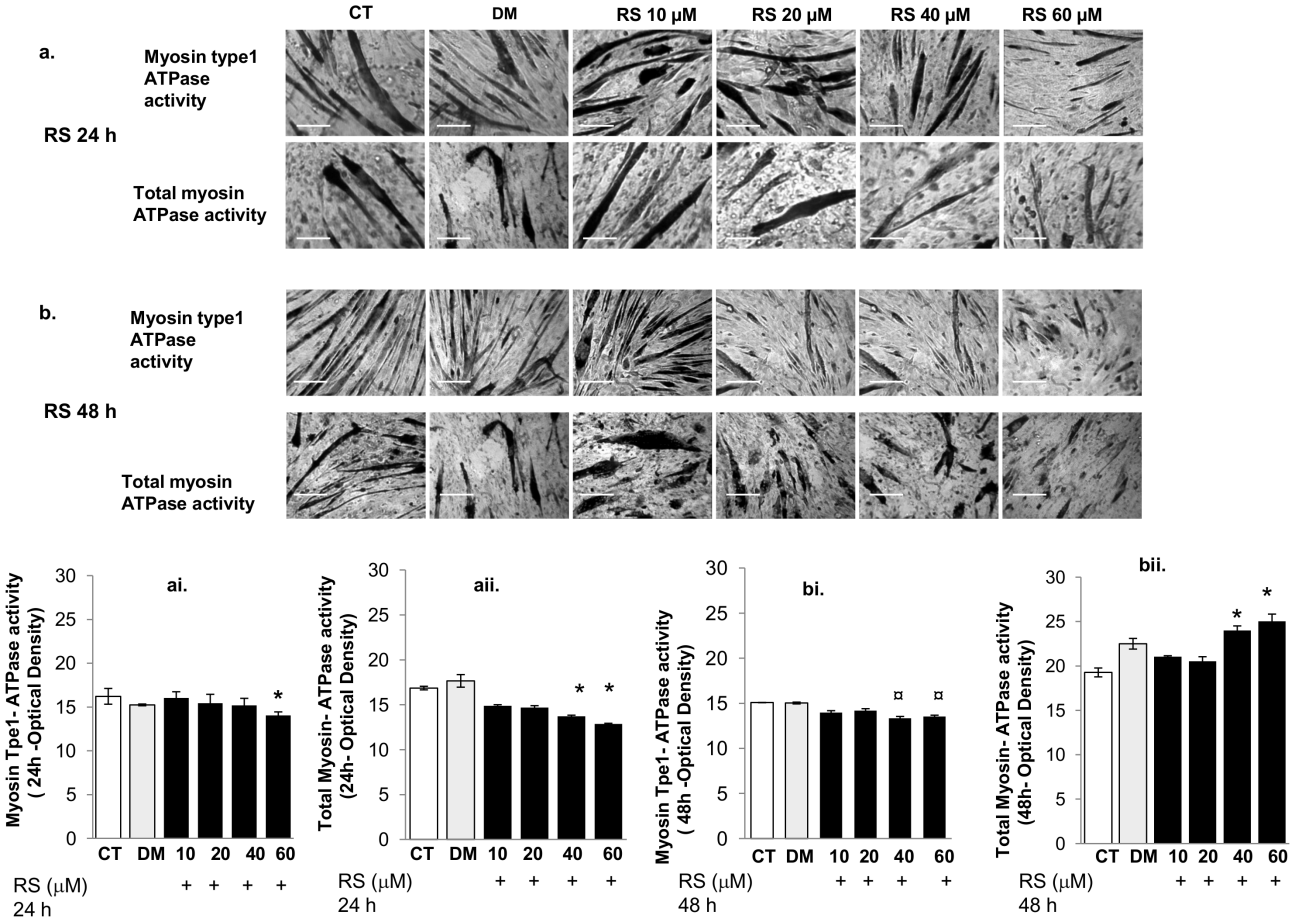
Resveratrol modulates myosin type 1 and total myosin ATPase activity. Representative phase contrast images showing the effect of 24 h (a) and 48 h (b) of 10, 20, 40 and 60 µM resveratrol (RS) on intracellular myosin type 1 and total myosin (type 1 plus type 2) ATPase activity in C2C12 myotubes after 8 days in differentiation media (2% FBS). RS 10–20 µM did not significantly affect myosin ATPase activity either after 24 h (ai;aii) or 48 h (bi;bii). Forty and 60 µM RS caused a transient decrease in both type 1 (60 µM) and total (40 and 60 µM) myosin ATPase after 24 h incubation (ai;aii) and an increase in total myosin (bii), but not type 1 myosin (above normal levels) after 48 h incubation. *P*-values calculated using a two-tailed Student's *t*-test.*: *P*<0.01 vs CT; ¤: *P*<0.05 vs CT. DM vs. CT: in none of the cases significant. Bars 20 µm. Original magnification 100×. Data are expressed as mean ± s.e.m. of biological triplicates.

To gain more information about the effect of RS on the properties of myofibrils, we tested the effect of different concentrations of RS on myosin type 1- and total myosin ATPase activities in C2C12 myotubes (Fig. 4). The effects of RS (10–60 µM) were followed for 24 h (Fig. 4a) and 48 h (Fig. 4b) to establish the dose- and time-dependent response to the treatment.

Ten and 20 µM RS treatment had no significant impact after both 24 h or 48 h of incubation (Fig. 4a,b). Higher doses (40–60 µM) caused a decrease in type 1 and total myosin ATPase activity after both 24 and 48 h incubation. The total myosin ATPase activity was, however, only transiently reduced after 24 h incubation with 40–60 µM RS (Fig 4bi) and even elevated above normal levels after 48 h incubation (Fig. 4b).

### Effects of H_2_O_2_ with and without resveratrol

#### Resveratrol (10 μM) prevented the deleterious action of H_2_O_2_ on cell migration but not cell fusion

**Figure 5:**
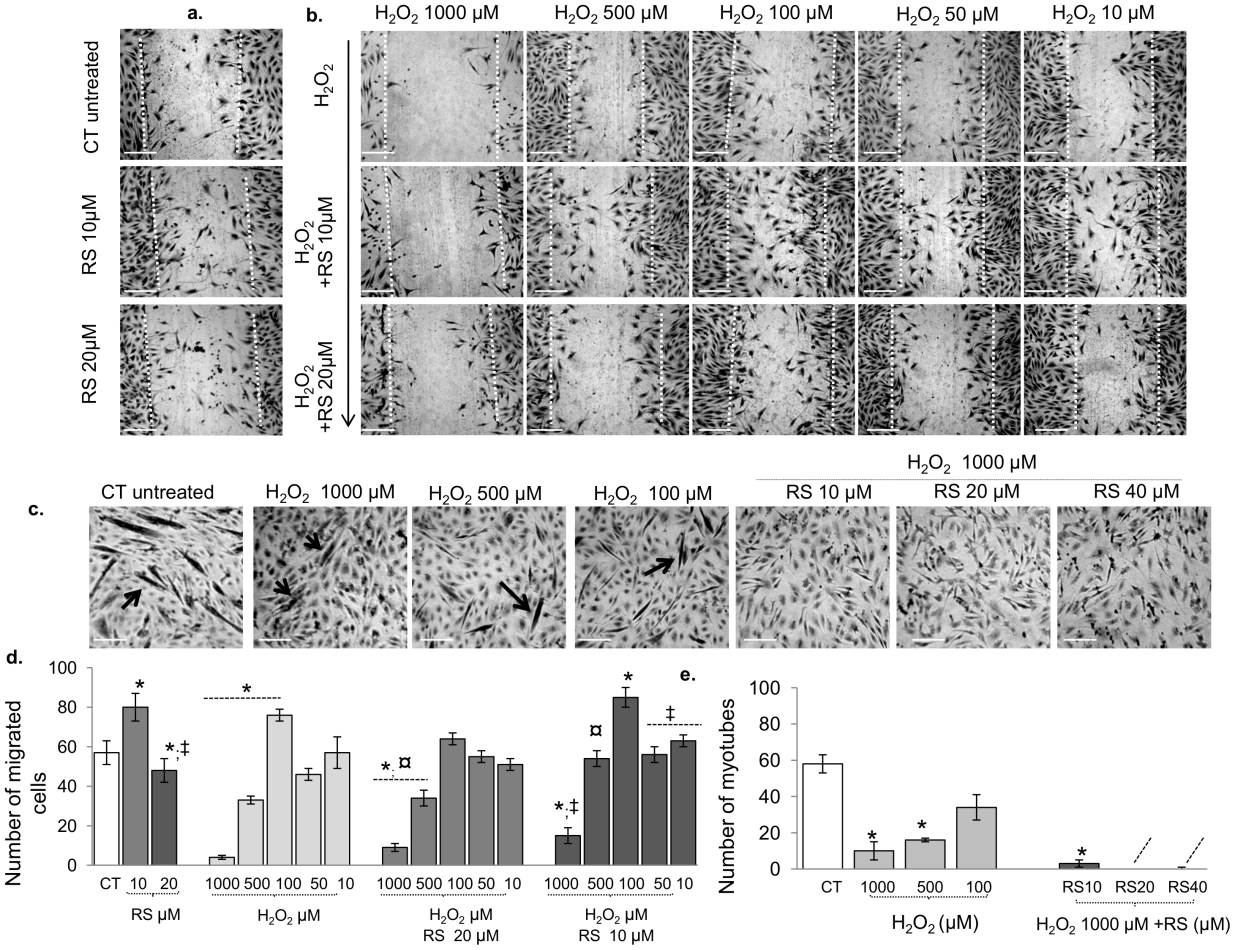
Resveratrol (10 µM) prevented the deleterious effect of H_2_O_2_ on cell migration but not cell fusion. (a) Phase contrast images showing the effect of RS alone (10 and 20 µM) on cell migration (24 h). (b, c) Phase contrast images showing the effects of different pre-conditioning resveratrol (RS) concentrations and H_2_O_2_ on cell migration (b) and fusion (c). The number of migrated cells and myotubes in the different conditions are summarized in (d) and (e), respectively. (b and graph in d) show that the effects of 24 h H_2_O_2_ on cell migration were dose-dependent. High doses of H_2_O_2_ (500–1000 µM) blocked cell motility, while it was increased with 100 µM H_2_O_2_. (b and graph in d) shows that RS pre-conditioning (10 µM) abolished the deleterious effects of 500 µM, attenuated that of 1000 µM H_2_O_2_ and further enhanced cell migration induced by H_2_O_2_ 100 µM. (c,e) 500 and 1000 µM H_2_O_2_ did block cell fusion, which was not rescued with RS preconditioning. Data are expressed as mean ± s.e.m of biological quadruplicate. *: *P*<0.01 vs. CT; ‡: *P*<0.01 vs. RS 10 µM; ¤: *P*<0.01 vs. RS 20 µM. *P*-value calculated using a two-tailed Student's *t*-test. Bars 20 µm. Original magnification, x 50.

Our second objective was to establish the optimal RS concentration to counteract or synergise the effects of H_2_O_2_ on cell migration and cell fusion (Fig. 5). We found that the effects of 24 h H_2_O_2 _on cell migration were dose-dependent (Fig. 5b,d). High doses of H_2_O_2 _(500 and 1000 µM) blocked cell motility almost completely, while it was increased by 100 µM H_2_O_2_, (Fig. 5b,d). High doses of H_2_O_2_ (500 and 1000 µM) also blocked cell fusion, as indicated by a reduced number of myotubes (Fig. 5c,e). Notably, 24 h of 10 µM RS pre-conditioning abolished the deleterious effects of 500 µM on cell migration and attenuated that of 1000 µM H_2_O_2._ In addition, 10 µM RS further enhanced cell migration induced by 100 µM H_2_O_2_. Twenty µM RS did not enhance the stimulating effect of 100 µM H_2_O_2 _nor have additional protective effects (Fig. 5b,d). Preconditioning with 10 µM RS for 24 h did, however, not rescue the inhibition on cell fusion induced by 1000 µM H_2_O_2 _(Fig. 5c,e).

After 24 h of treatment with 1000 µM H_2_O_2_ (Supplementary Fig. 2), we found a marked reduction of intracellular ROS in the surviving cells, which may be indicative of loss of mitochondria, either due to reduced biogenesis or mitochondrial destruction. This reduction in ROS was even more pronounced when the H_2_O_2_ cells were pre-treated with RS (Supplementary Fig. 2).

#### Resveratrol preserved myosin ATPase activity in C2C12 myotubes from H_2_O_2_ action

**Figure 6:**
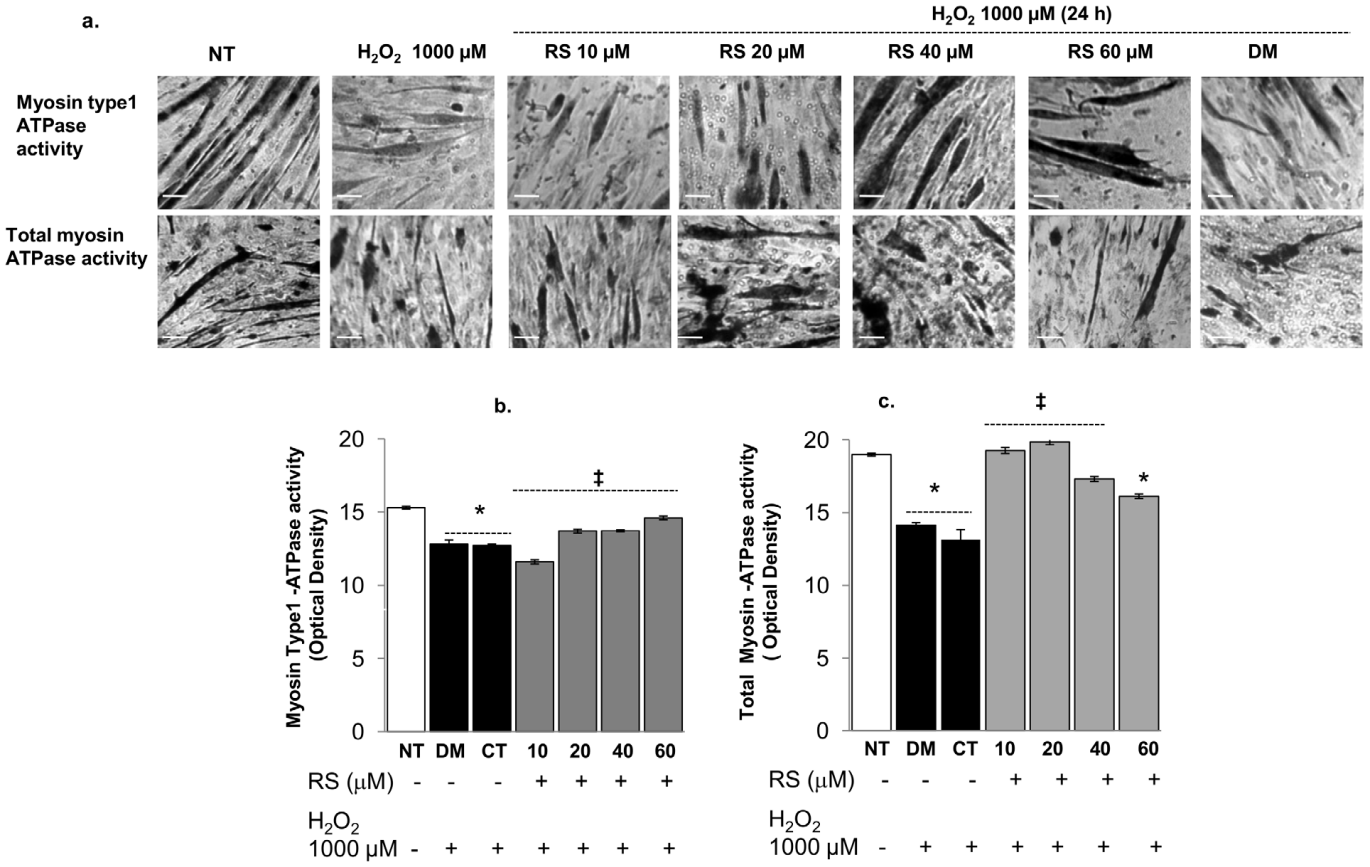
Resveratrol attenuated the impact of 1000 µM H_2_O_2_ on type 1 and total myosin ATPase activities in C2C12 myotubes. (a) Representative phase contrast images showing the effect of different concentrations of resveratrol (RS) preconditioning and 1000 µM H_2_O_2_ on myosin type 1 and total myosin ATPase activity in C2C12 myotubes that have been 8 days in differentiation media (2% FBS). H_2_O_2_ (1000 µM) did reduce both myosin type 1 (b) and total (c) myosin ATPase activities. Ten µM RS pre-conditioning further reduced the myosin type 1 activity (b), but abolished the impact of H_2_O_2_ on total ATPase activity (c). Higher RS concentrations did attenuate the impact of H_2_O_2_ on both myosin type 1 and total ATPase activity (b,c). DM+1000 µM H_2_O_2_ vs. CT+1000 µM H_2_O_2_ in none of the cases significant. Data are expressed as mean ± s.e.m. of biological quadruplicate. *P*-values calculated using two-tailed Student's *t*-tests. *: *P*<0.01 vs. NT (untreated); ‡: *P*<0.05 vs. 1000 µM H_2_O_2_. Bars 10 µm.

As shown in Figure 6, we found that H_2_O_2_ did significantly reduce myosin type 1 and total myosin ATPase activity. To test whether RS provides protection against the effects of H_2_O_2_ on myosin-ATPase activity, C2C12 myotubes were cultured and pre-conditioned (24 h) with different concentrations of RS and then treated for 24 h with 1000 µM H_2_O_2_. Pre-incubation with 10 and 20 µM RS did counteract the inhibitory effect of H_2_O_2_ on total ATPase activity (Fig. 6c), but did show only a marginal protective effect on myosin type 1 activity (Fig. 6b). Higher RS concentration did attenuate the impact of H_2_O_2_ on both myosin type 1 and total ATPase activity, with a more marked effect on total ATPase.

#### Resveratrol induced mitochondrial damage and was not sufficient to prevent mitochondrial membrane depolarisation in response to H_2_O_2_

**Figure 7:**
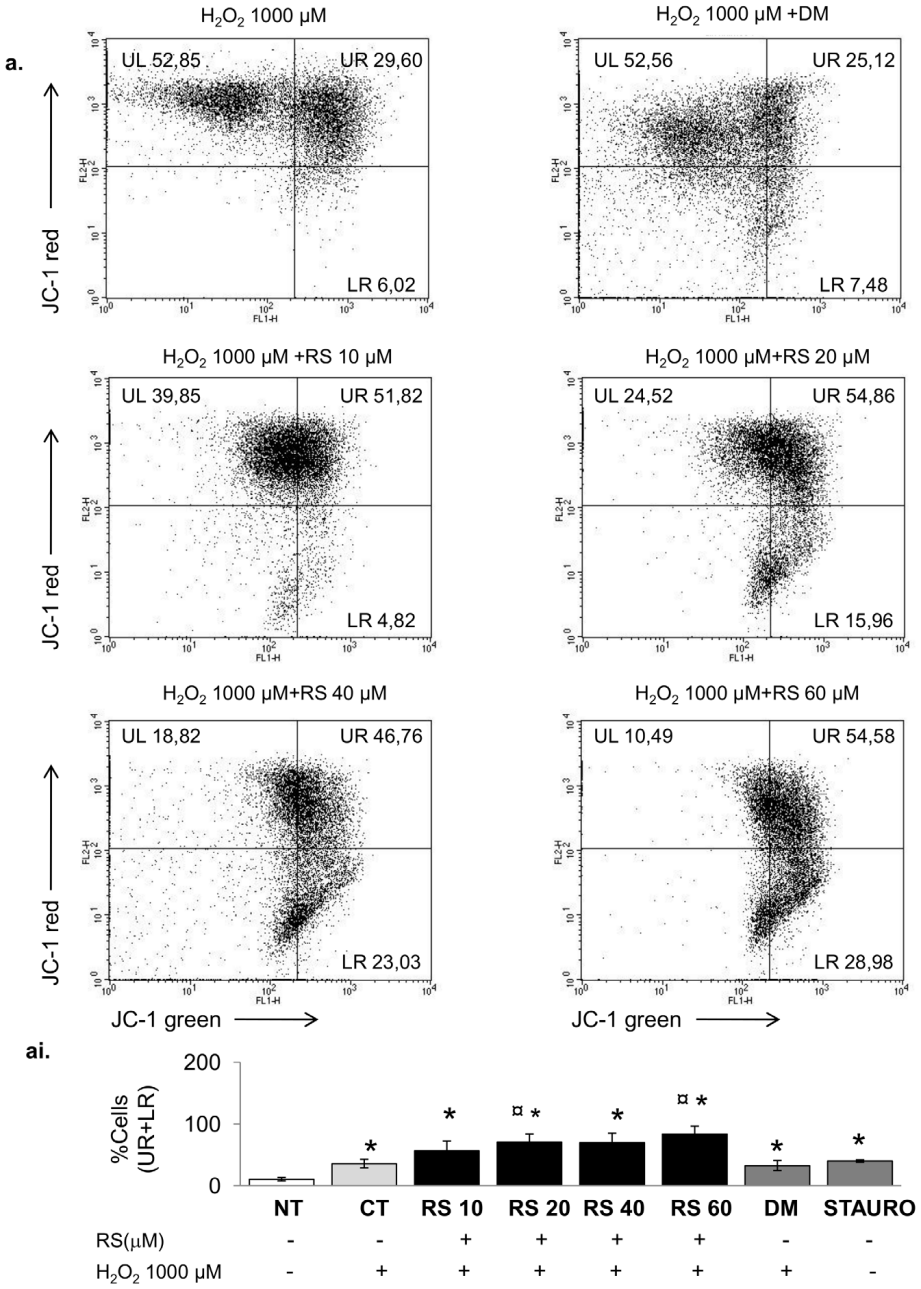
Resveratrol enhanced mitochondrial membrane depolarisation induced by H_2_O_2_. (ai) C2C12 myoblasts were exposed to 1000 µM H_2_O_2_ with or without RS preconditioning for 24 h as described in the text. Appropriate amounts of DMSO (DM pre-conditioning), followed by incubation with 1000 µM H_2_O_2_ (24 h), were used to ensure that the effects were resveratrol-specific. A positive control of mitochondrial depolarisation was obtained by two hours treatment with 5 µM of the apoptotic inducer staurosporine. The total % JC-1 green fluorescence cell population, including shift in depolarisation (gate UR+LR) was calculated. (a) representative images showing each condition and cell percentages in each gate from independent experiments. (aii) Percentage of depolarised cells (gate UR+LR) under different conditions. H_2_O_2_ with/without RS pre-conditioning induced mitochondrial depolarisation. Notably, with respect to H_2_O_2_ alone, RS pre-conditioning enhanced the effect of H_2_O_2_ toward the depolarised state (ai, LR panels), in a dose-dependent manner. DM+1000 µM H_2_O_2_ vs. CT+1000 µM H_2_O_2_ in none of the cases significant. Data are expressed as mean ± s.e.m. *P*-values calculated using a two-tailed Student's *t*-test. *: *P* = 0.01 vs NT (untreated); ¤: *P*<0.05 vs H_2_O_2_ 1000 µM. Each experiment was performed in quadruplicate.

**Figure 8:**
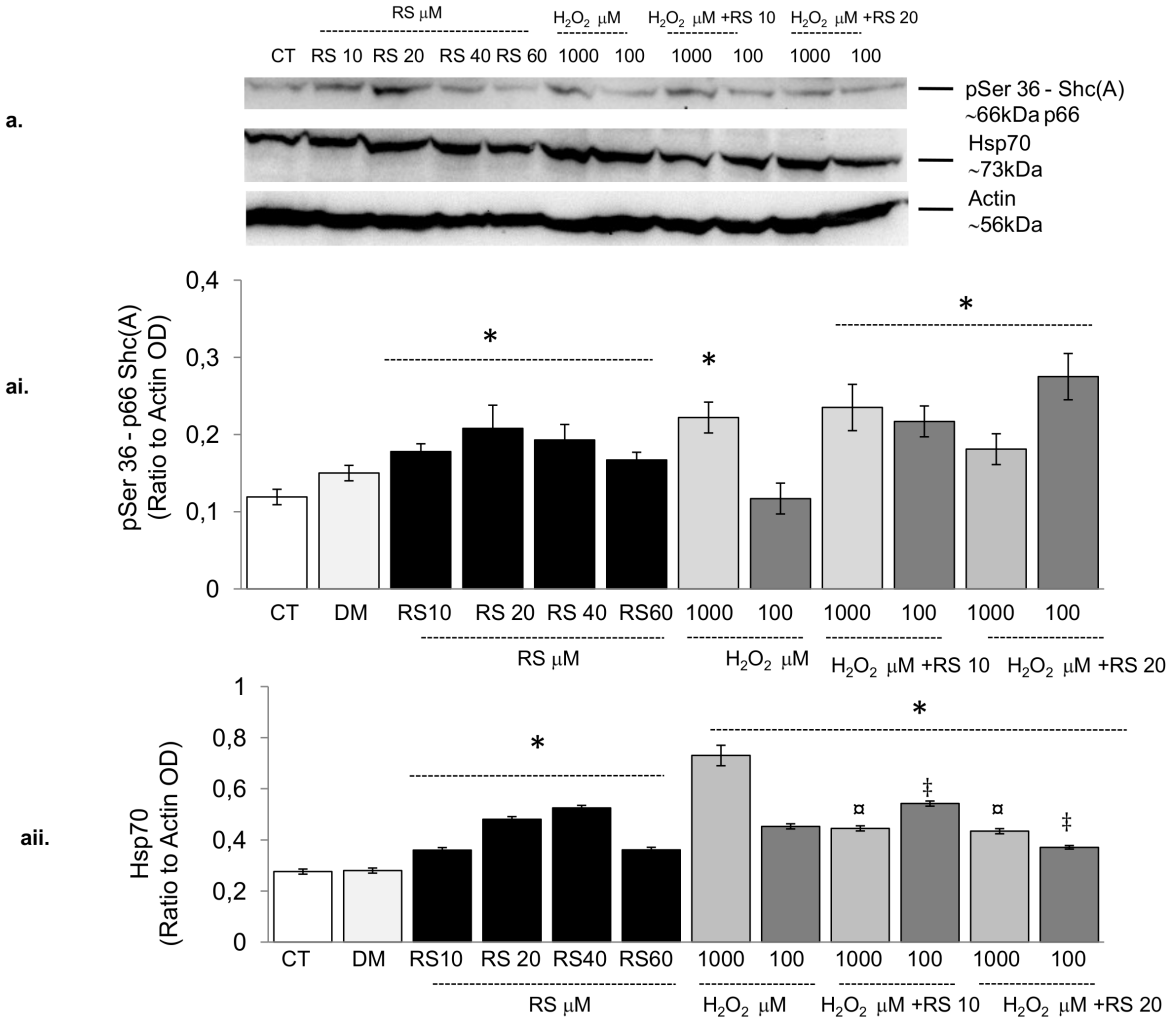
Resveratrol induced p66Shc-Ser36 phosphorylation and up-regulation of Heat Shock Protein (Hsp)-70. (a) Western blot analysis showing the effect of resveratrol (RS; 10, 20, 40 and 60 µM), H_2_O_2_ (100 and 1000 µM) and RS pre-conditioning (10 and 20 µM) on p66Shc-Ser36 phosphorylation and Hsp-70 protein levels. Dose dependent increases in Ser36-p66 phosphorylation and in Hsp-70 protein contents were observed in resveratrol-treated cells. 1000 µM, but not 100 µM H_2_O_2_ induced p66Shc(A)-Ser36 phosphorylation (ai); both concentrations of H_2_O_2_ elevated Hsp-70 protein levels, but more so in 1000 than 100 µM H_2_O_2_ (aii). RS (10 and 20 µM) pre-conditioning attenuated the effects of 1000 µM H_2_O_2_ on Hsp-70 (aii), but not on p66Shc-Ser36 phosphorylation (ai). DM vs. CT: in none of the cases was significant. *P*-values calculated using a two-tailed Student's *t*-test.*: *P*<0.01 vs CT; ¤: *P*<0.001 vs H_2_O_2_ 1000 µM; ‡: *P*<0.01 vs H_2_O_2_ 100 µM. Data are mean ± s.e.m.

The effects of H_2_O_2_ on mitochondrial membrane potential (ΔΨm) and its implication in the activation of the mitochondrial apoptosis cascade are well recognized^37^. Resveratrol has been suggested to counteract apoptosis induced by H_2_O_2_. Therefore, we analysed whether RS was able to counteract H_2_O_2_-induced mitochondria depolarisation. RS pre-conditioning did not prevent, but even enhanced the depolarization induced by 24 h exposure to 1000 µM H_2_O_2_ (Fig. 7). Notably, this was associated with increased phosphorylation of p66Shc(A)-Ser36 (Fig. 8ai), and elevated Hsp-70 protein levels (Fig. 8aii), two key players in the mitochondrial and cellular stress response^37^,^38^. In particular, 1000 µM, but not 100 µM-H_2_O_2_ induced p66Shc(A)-Ser36 phosphorylation (Fig. 8ai) and both elevated Hsp-70 protein levels (Fig. 8aii). Although pre-incubation with RS reduced the 1000 µM H_2_O_2_-induced elevation in Hsp70, it did not alleviate the increased p66Shc(A) phosphorylation.

#### Resveratrol further enhanced the inhibitory effect of H_2_O_2_ on energy metabolism

C2C12 myoblasts (for active metabolic state; Fig. 2b) and myotubes (for SDH activity; Fig. 3b) were cultured 24 h and 48 h in the absence or presence of RS scalar amount, or vehicle. RS preconditioning was performed by 24 h of treatment with RS scalar amount (day1) then followed by 24 h of treatment with 100, 500 and 1000 µM H_2_O_2_ (day2). Only the highest concentration of H_2_O_2_ caused a reduction in both metabolism in myoblasts (Fig. 2b) and oxidative capacity in myotubes (Fig. 3b), which was further decreased by pre-treatment with increasing doses of RS (Fig. 2b and Fig. 3b).

## Discussion

Muscle repair is a complex process that requires the coordination of several steps, starting with the activation of muscle satellite cells, to continue with myoblast proliferation, migration and withdrawal from the cell cycle^1^. The final step is the fusion and subsequent differentiation of the satellite cell with the damaged fibre^1^. Here we have analysed the effects of resveratrol on different steps of *in vitro* muscle regeneration, looking at myoblast proliferation, cell cycle progression, cell motility, cell fusion and sprouting. In addition, we studied the potential of resveratrol to modulate muscle remodelling in relation to different degrees of superimposed exogenous H_2_O_2_ as model of oxidative stress.

Our main observations were that the effects of resveratrol on *in vitro* muscle cell plasticity were dose dependent and also dependent on the degree of exogenous oxidative stress. Low doses stimulated cell cycle arrest, cell migration and sprout development, while higher concentrations blocked the regenerative process almost completely and even showed a marked cytotoxic effect. However, resveratrol at low concentrations (10 µM) did show only a modest effect in attenuating the detrimental impact of high doses of H_2_O_2_.

### The effects of Resveratrol on cell viability

The literature is equivocal when it comes to the effectiveness of RS to improve muscle contractile function and metabolism. It has been reported for instance that RS has positive effects on muscle mass and function^14^,^19^,^26^,^39^,^40^, increases fibre oxidative metabolism, mitochondrial function and muscle aerobic capacity in rodents^39^,^40^, improves muscle mass recovery during reloading^26^, alleviates oxidative stress in aged mice^13^ and reduce muscle cell death^19^. Others, however, did not find beneficial effects of RS^24^,^25^,^27^,^41^,^42^,^43^, or even report toxic effects of RS on mitochondria and cells^31^,^32^.

Several authors indicated that RS promotes the early stage of C2C12 myoblast differentiation^23^,^44^ by inducing the expression of transcription factors and differentiation markers after 24 h incubation. In support of this, we found that 10 µM RS induced cell cycle arrest and improved the quality of cell sprouting. Yet, despite the cell cycle arrest, we did not find an increase in myotube formation in myoblasts treated with 10 µM RS (Fig. 1). It is possible that 10 µM RS inhibits proliferation and promotes the early stages of differentiation, but does not stimulate the late phase of myoblast differentiation. With higher RS concentrations (>20 µM) there was even evidence for enhanced cell cycle progression (increased proportion of cells in the G2+S phases) and inhibition, rather than stimulation, of differentiation as reflected by an almost completely blocked sprout formation and myoblast cell fusion (Fig. 1). Clearly, the effects of RS on differentiation and proliferation are dose dependent.

It has recently been demonstrated that high RS concentrations (>30 µM) impair cell viability^30^,^31^,^32^,^42^,^43^,^44^. In line with this, we found that high doses (>20 µM) of RS diminished cell viability (Supplementary Fig. 3b). The decreased cell viability might have a mitochondrial origin. Although some studies have shown beneficial effects of RS on mitochondrial function^39^, it has also been reported that the simultaneous inhibition of NADH:ubiquinone oxidoreductase and F0F1-ATPase/ATP synthase^42^,^43^, and the accumulation of RS metabolites in the mitochondria^32^ significantly impair mitochondrial function. This in turn would cause decreased ATP levels, mitochondrial membrane depolarisation, and generation of reactive oxygen species and induction of apoptosis^43^. It is important to underline, that the degree of cytotoxicity depends on the cell type, organism and/or the dose and duration of exposure to RS^33^. Here we found that the mitochondrial membrane depolarisation (Supplementary Fig. 3a), a key trigger of apoptosis^45^, was increased with increasing doses of RS and associated with an increased percentage of apoptotic/necrotic myoblasts (Supplementary Fig. 1c) and reduced cell viability (Supplementary Fig. 3b). Notably, RS induced a cellular stress response, as indicated by the increased level of the heat shock protein-70 protein and phosphorylation of the stress response protein p66Shc (A), a key player in mitochondrial depolarisation and oxidative stress^37^,^45^ (Fig. 8). Thus, the dose-dependent reduction in cell number after RS incubation (Fig. 1b), may be the result of both a reduction in cell proliferation, as a consequence of cell cycle arrest, and increased cell death, as a consequence of mitochondrial dysfunction.

The depolarisation of mitochondria may well lead to loss of mitochondria in the cell. In myotubes, the reduced succinate dehydrogenase (SDH) activity, which plays a role in both the citric acid cycle and the respiratory chain, and total mitochondrial dehydrogenase activity after exposure to high doses of RS, indicates that this is indeed the case (Fig. 3). A lower SDH activity has also been suggested to be an important hallmark of mitochondrial dysfunction and reduced cell viability^35^.

The reduced ROS generation in myoblasts incubated with RS may at first glance fit the notion that RS is an effective anti-oxidant. Given the observations discussed above, a more likely explanation is that the RS-induced loss of mitochondria underlies the reduction in ROS production.

Finally, high doses of RS reduced myosin type1-ATPase activity (Fig. 4) to enhance that of total myosin ATPase, suggesting an increase in myosin type-2 (Fig. 4). Such a slow-to-fast transition in the myosin heavy chain composition *in vivo* would cause a reduced fatigue resistance of the muscle.

Thus, although several *in vivo* studies report the potential of RS to diminish mitochondrial oxidative injury and cell death of skeletal muscle cells^19^, our results show that at higher doses RS is detrimental rather than beneficial for C2C12 regeneration and mitochondrial function. However, we did not directly quantify the effect of resveratrol on some classical markers of the endogenous anti-oxidant defence (i.e. catalase or superoxide dismutase1). Such information could clarify whether RS could induce an excess of ROS scavenging, as suggested previously by *in vivo* work^13^, that would convey over time a reduction in oxidative stress and improve cell function^7^,^13^. Here we observed a RS dose-dependent increase in protein levels of the cytoprotective and anti-oxidant protein Hsp70^46^ (Fig. 8aii), that may suggest activation of ROS scavenging, that ultimately would reduce cellular oxidative stress^47^. Some support of this is seen in the diminished SDH activity in myotubes after 24 h incubation with RS that was normalised after 48 h of incubation (Fig. 3).

### The impact of H_2_O_2_ with and without resveratrol on cell viability

In line with the reported beneficial effects of low and moderate levels of ROS^8^, we observed that 100 µM H_2_O_2_ stimulated *in vitro* cell motility (Fig. 5). At higher doses (500 & 1000 µM H_2_O_2_) *in vitro* cell motility was inhibited. Only low doses (10 µM) of pre-conditioning with RS for 24 h attenuated the deleterious impact of high ROS exposure on cell migration (Fig. 5) and even enhanced cell motility induced by moderate doses of H_2_O_2_, while higher doses of RS doses did not show any effect.

Part of the impaired motility may be consequent to mitochondrial dysfunction and accompanying impairments in mitochondrial ATP synthesis. In line with this, we observed in myoblasts that the mitochondrial membrane depolarisation induced by 1000 µM H_2_O_2_ was further aggravated with RS-preconditioning (Fig. 5). As discussed above, the reduction in ROS after H_2_O_2_ incubation with or without RS (Supplementary Fig. 2) is most likely due to loss of mitochondria, as reflected by the reduced SDH activity of the incubated myotubes (Fig. 3). While RS pre-conditioning partially prevented the increase in Hsp70 induced by 1000 µM H_2_O_2_, it did not rescue the pSer36 phosphorylation (Fig. 8ai), suggesting that the stress response after RS preconditioning was even worse, and may have contributed to the more pronounced loss of mitochondria.

### Conclusion

In conclusion, our study shows that low doses of RS may be beneficial, but high doses of RS are detrimental. We found that high doses of RS could even aggravate the consequences of oxidative stress. More specifically, low doses of resveratrol stimulated cell migration, cell cycle arrest and cell sprouting, while similar to ROS, high doses of RS had detrimental effects on muscle cell viability, mitochondria stability and muscle oxidative capacity. To our surprise, none of the RS concentrations improved the oxidative capacity or metabolic capacity of myotubes.

## Methods

### Mouse C2C12 cell culture and treatments

Murine C2C12 myoblasts (American Type Culture Collection, ATCC; Rockville, MD, USA) were cultured on 0.2% gelatin (Sigma,-Aldrich, Germany) coated T75 flasks and/or plates in Dulbecco's modified Eagle's medium (DMEM; Clonetics, Lonza, Germany) in a 37°C humidified chamber at 5% CO_2_. The medium was supplemented with 10% heat-inactivated fetal bovine serum (FBS; Gibco, UK), 2 mM L-glutamine and 1% penicillin-streptomycin solution (Invitrogen, UK). At 70% confluence, the cells were divided by trypsinisation (0.5% trypsin in 0.5 mM EDTA; Sigma-Aldrich, Germany). DMEM supplemented with 2% FBS, was used as differentiation medium^48^. DMEM, supplemented with 0.1% FBS, was used to minimize proliferation during migration assays^49^. Experiments were performed for 24 or 48 h in the presence or absence of (10, 20, 40 or 60 µM) resveratrol (RS; 98.57% pure, *Poly-gonumcuspidatum* extract; 21^st^ Century Alternative, UK), and/or 10, 50, 100, 500 and 1000 µM hydrogen peroxide (H_2_O_2_; Sigma-Aldrich, Germany). The chosen ranges of concentration of RS and H_2_O_2_ were based on studies demonstrating the effectiveness of similar concentrations^10^,^28^,^48^,^50^ Appropriate amount of Dimethyl sulfoxide (DMSO), the resveratrol vehicle, was tested alone to ensure that the effects were resveratrol-specific. The final DMSO concentration in the media did not exceed 1.0%^48^.

### *In vitro* C2C12 cell proliferation assay

Myoblasts were seeded in complete DMEM medium at a concentration of 2 × 10^5^ cells·mL^−1^ (2 mL per well) in 0.2% gelatin-coated 6-well plates. After attachment (4 h) cells were washed twice with sterile phosphate buffered saline (PBS, pH = 7.4) and the medium replaced with complete DMEM containing 10, 20, 40 or 60 µM RS and/or DMSO. Cells were counted after 24, 48 or 72 h incubation with an automated Coulter counter (Coulter Electronics, Hialeah, FL). Each experiment was performed in triplicate.

### Cell cycle and cell viability

Cell cycle analysis was performed as described in^48^,^51^. Cell viability was assessed with the colorimetric assay CellTiter 96® AQueous One Solution Cell Proliferation Assay MTS (Promega, UK). Quantification of apoptotic cells was carried out by measurement of sub-G1 DNA content using propidium iodide^52^. After 24, 48 or 72 h incubation in complete DMEM, adherent cells were trypsinized in 1.5 mL of 0.5% trypsin–0.02% EDTA, and pooled with detached cells. They were then suspended in 5 mL complete media, centrifuged (300 *g,* 10 min, 4°C) and washed in PBS prior to fixation at −20°C in 70% ethanol. Twenty four hours later the fixed cells were recovered by centrifugation, washed in PBS and suspended with gentle vortexing in propidium iodide labelling buffer (50 *μ*g·mL^−1^ propidium iodide and 20 *μ*g·mL^−1^ ribonuclease A (Sigma Aldrich, Germany), at approximately 1 × 10^6^ cells·mL^−1^ 51. Cells were then stored in the dark at 4°C for 30 min and analyzed at room temperature using a FACSCalibur^TM^ flow cytometer (Becton Dickinson, Oxford, UK). The fluorescence data (FL-H channel) on 10,000 event counts were analysed using Cell Quest (Becton Dickinson, UK) and Modfit LT Software (Verity Software, Topsham, ME, USA). A coefficient of variation of the G1 peak <6 represented acceptable quality data^52^. All experiments were performed in triplicate.

### *In vitro* morphological differentiation

To induce differentiation, myoblasts were grown to about 50% confluence^48^. The growth medium was then replaced with DMEM (2% FBS; differentiation medium) with the presence or absence of scalar amounts of resveratrol, H_2_O_2_ or vehicle. The effect of the compounds on cell fusion was determined after 72 h by counting the total number of 1% methylene blue stained myotubes (being defined as having at least three nuclei within one cytoplasmatic continuity) present in three random picture fields captured from each well with an inverted microscope. All experiments were performed in triplicate.

### *In vitro* spheroid based myotube analysis

The C2C12 spheroids assay was performed to test the effect of scalar amounts of resveratrol on cell sprouting^53^, seen as the formation of elongated extensions on spheroids. Briefly, cells were harvested from sub-confluent monolayer cultures by trypsinisation and 6 × 10^5^ cells·mL^−1^ were suspended in DMEM plus 2% FBS and 0.25% (w/v) carboxymethylcellulose (Sigma-Aldrich, Germany). After their formation (24 h), single independent spheroids where sub-cultured for 24 h at 37°C, 5% CO_2_ with/without the presence of scalar amounts of resveratrol and/or vehicle in a matrix of type I collagen (BD Bioscence, UK)^53^. After 24 h, sprouts formed from single independent spheroids were photographed (Nikon inverted microscope)^53^. The number of sprouts and their length were then analysed and quantified using ImageJ 1.47software (rsbweb.nih.gov/ij/). All experiments were performed in triplicate.

### *In vitro* cell migration in wound healing

C2C12 myoblasts (1–2 × 10^4^·mL^−1^) were added to a 0.2% gelatin-coated 24-well plate in complete DMEM medium. After attachment (4 h) cells were washed twice with PBS and the medium replaced with DMEM (0.1% FBS) and maintained at 37°C and 5% CO_2_ for 24 h to minimize cell proliferation. Migration assay^49^,^53^ was then performed in the presence or absence of scalar amounts of resveratrol and/or H_2_O_2_ or vehicle (DMEM 0.1%) and followed for 24 h. In the experiment of RS pre-conditioning, cells were pre-treated (24 h) with (10 or 20 µM) RS and migration assays (24 h) performed with scalar amount of H_2_O_2_ (DMEM 0.1%). All experiments were done in triplicate.

### Intracellular levels of reactive oxygen species (ROS)

After each treatment, 1 × 10^6^ ·mL^−1^ myoblasts were incubated for 1 h at 37°C with the cell permeable fluorescent and chemiluminescent probes 2′-7′-Dichlorodihydrofluorescein diacetate (DCFH-DA; Invitrogene, UK)^54^ and ROS levels detected by flow cytometer analysis^54^ (FACSCalibur^TM^, Becton Dickinson, Oxford, UK) and CellQuest software (BD Biosciences). For the RS pre-conditioning, cells were pre-treated (24 h) with 10 or 20 µM RS, followed by 24 h treatment with 1000 µM H_2_O_2_. Before each treatment, cells were washed twice with sterile PBS. All experiments were performed in triplicate.

### Flow cytometric analysis of mitochondrial membrane depolarisation

Myoblasts were cultured (24 h) in the presence or absence of scalar amounts of RS, H_2_O_2_ and/or vehicle. After treatment, ~1 × 10^6^ cells·mL^−1^ were incubated for 30 min at 37°C with 5 µg·mL^−1^ final concentration of the cationic dye 5,5′,6,6′-tetrachloro-1,1′,3,3′ tetraethylbenzimidazolyl-carbocyanine iodide (JC-1; Invitrogene, UK), with conditions as described by the manufacture. Analyses were then performed by flow cytometry^55^ (FACSCalibur^TM^ flow cytometer, Becton Dickinson, Oxford, UK) and CellQuest software (BD Biosciences). Cells (10,000 count events) were analysed by FL-1 (green fluorescence) and FL-2 (red fluorescence) channels. Gates (*Upper Left*-polarised mitochondria, UL; *Upper Right*-mixed cell population i.e. polarised and depolarised mitochondria, UR; Lower Right-depolarised mitochondria, LR) were established with the untreated cells (controls)^55^. Calculations were performed, considering the total % of JC-1 green fluorescence cell population (gates UR+LR; depolarised cells) under different conditions. All experiments were performed in quadruplicate.

### *In vitro* mitochondrial dehydrogenases activity

The activity of mitochondrial dehydrogenases was measured by a colorimetric assay (CellTiter 96® AQueous One Solution Cell Proliferation Assay MTS, Promega, UK), based on the redox conversion of a tetrazolium salt into a formazan product^56^. In the experiments of RS pre-conditioning, cells were pre-treated (24 h; day1) with scalar amounts of resveratrol, followed by 24 h of H_2_O_2_ (day2). Treatment with H_2_O_2_ alone was also performed at day2. Twenty-four hours after incubations, 5 × 10^3^ cells·mL^−1^ were seeded in complete DMEM medium on 0.2% gelatin-coated 96-well plates. After attachment (4 h) cells were washed twice with PBS and the medium replaced with complete DMEM containing the tetrazolium salt and incubated at 37°C for 4 h. The absorbance of the formazan was read at 490 nm. Data from H_2_O_2_ alone and RS preconditioning were compared to control conditions at day2. All experiments were performed in quadruplicate.

### *In vitro* Myosin-ATPase assay

C2C12 myoblasts were cultured in reduced DMEM media (2% FBS) for 8 days. Occasionally, a spontaneous contractile activity was observed at the 8^th^ day, suggesting functional maturity^10^. Then, myotubes underwent 24 and/or 48 h of treatment with scalar amounts of RS and/or H_2_O_2_ or vehicle. In the RS-preconditioning experiments, cells were pre-treated (24 h) with resveratrol and/or vehicle. After the treatments, the media was removed and cells were stained for myosin ATPase as described^57^. Briefly, total myosin ATPase activity was reflected by intracellular myosin ATPase staining after a pre-incubation at pH = 9.4; myosin type-1 that after a pre-incubation at pH = 4.3^57^. Myosin ATPase activity was given as the optical density of each single stained cell present in eight random picture fields, captured by a Nikon inverted microscope. Quantitative analysis was performed by ImageJ software; data were expressed in grey scales. All experiments were performed in triplicate.

### *In vitro* myotube succinate dehydrogenase (SDH) activity

C2C12 myoblasts were cultured in DMEM (2% FBS) for 8 days. At the 8^th^ day, they were treated 24 or 48 h with RS (10, 20, 40, 60 µM) and/or 24 h with scalar amount of H_2_O_2_ (100, 500 or 1000 µM) or vehicle. In the RS-preconditioning experiments, cells were pre-treated (24 h; day1 corresponding to the 9^th^ day of myoblast cultured in differentiation media) with resveratrol or vehicle. After two washes with PBS, the media was removed and myotubes were cultured for another 24 h (day2 corresponding to the 10^th^ day of myoblasts cultured in differentiation media) in the presence of 1000 µM-H_2_O_2_. Staining for SDH activity was performed as described previously^57^. SDH activity was then determined from eight random picture fields captured from each experimental well and analysed by ImageJ software; and reported as the optical density of SDH. All experiments were performed in triplicate.

### Western Blotting

Protein extraction was performed in ice-cold RIPA buffer with a protein inhibitor cocktail. Thirty μg protein was loaded and separated on 10% SDS-PAGE gels. Protein samples were transferred onto nitrocellulose membranes (Whatman International Ltd.), stained with amido-black and photographed to verify equal loading and quality of the transfer. The membranes were subsequently incubated with blocking solution and incubated overnight at 4°C with primary antibodies against mouse p66Shc-pSer36 (Calbiochem, UK), heat shock protein 70 (Abcam, UK) or mouse actin (Sigma-Aldrich, Germany). The membranes were then incubated with appropriate horseradish-peroxidase-conjugated secondary antibodies (Dako, UK) at room temperature. Protein bands were visualised by chemiluminescence detection kit (Gibco®, Invitrogene Life Science, UK) and signals normalized to the corresponding actin optical density (Bio-Rad quantity one software, UK).

### Statistical analysis

All data were expressed as mean ± s.e.m. Effects of the treatments were assessed using two-tailed Student's *t*-test or ANOVA (SPSS version 12) and considered significant at *P*<0.05. Experiments were performed in triplicate or quadruplicate as described in Methods.

## Author Contributions

A.B. and H.D. conceived the study. A.B. designed and performed the experiments. A.B. and H.D. analysed the data and wrote the manuscript.

## Supplementary Material

Supplementary Information Supplementary Figures and supplementary figure legends
