# Supplementary material for: The impact of resveratrol and hydrogen peroxide on muscle cell plasticity shows a dose-dependent interaction
Source: Sci Rep. 2015 Jan 28;5:8093. doi: 10.1038/srep08093 (PMC4308712; doi:10.1038/srep08093)
Supplement: Supplementary Information — Supplementary Figures and supplementary figure legends [file srep08093-s1.doc]

**Supplementary Information**

**The impact of resveratrol and hydrogen peroxide on muscle cell plasticity shows a dose-dependent interaction**

Alessandra Bosutti^1,a^,* & Hans Degens^1^,*

^1^School of Healthcare Science, Manchester Metropolitan University, Manchester, United Kingdom

^a^ Present affiliation: Department of Medical, Surgery and Health Sciences, University of Trieste, Trieste, Italy

*Address for correspondence:

Dr. Alessandra Bosutti

Department of Medical, Surgery and Health Sciences, University of Trieste, c/o Cattinara Hospital, Strada di Fiume 447

e-mail: [bosutti@units.it](mailto:bosutti@units.it)

Dr. Hans Degens:

School of Healthcare Science, Manchester Metropolitan University, John Dalton Building; Chester Street, Manchester M1 5GD , United Kingdom

e-mail: h.degens@mmu.ac.uk

*Supplementary Figure 1*

**
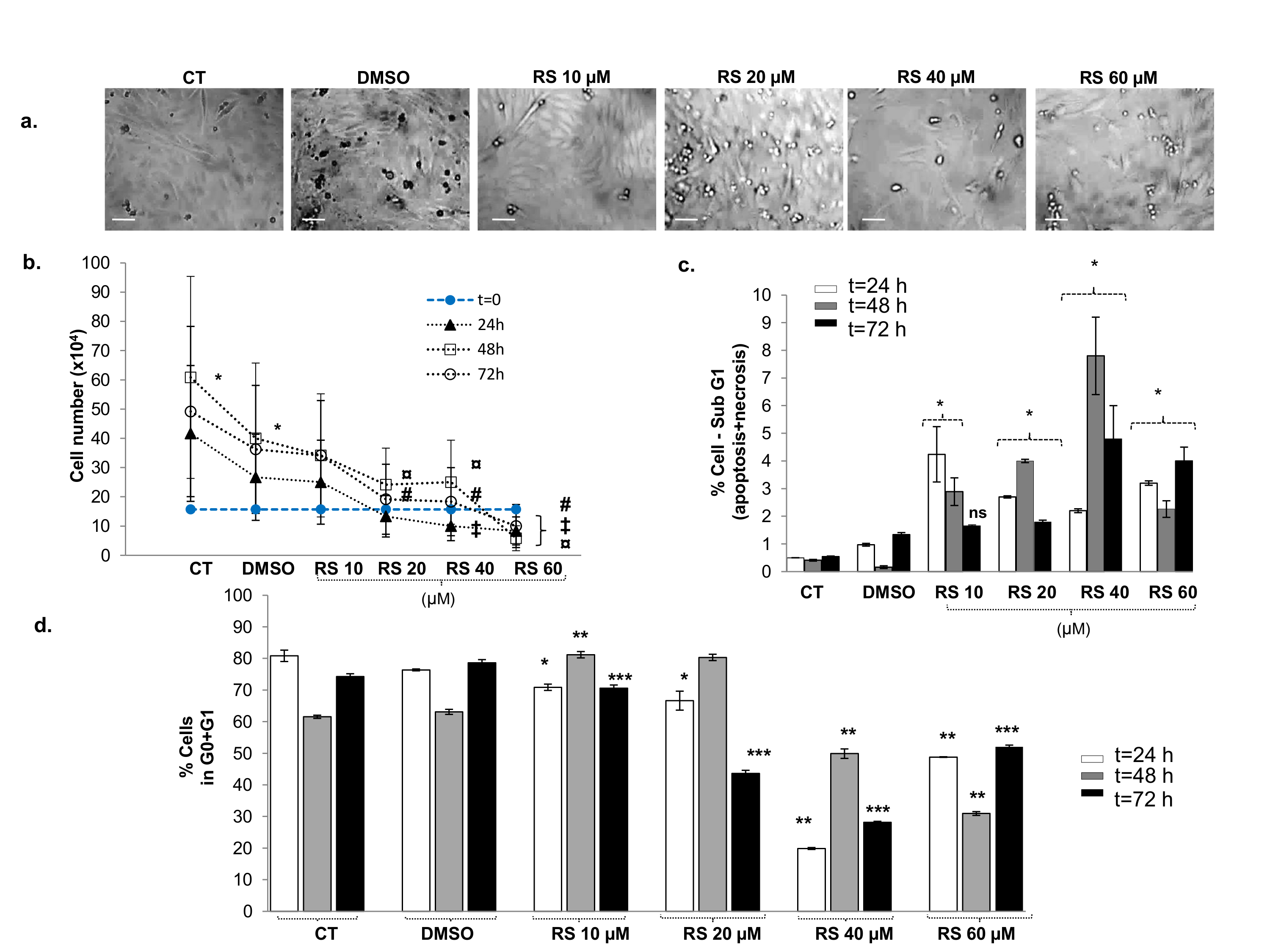
**

*(Supplementary Figure 1) Panel e*

**
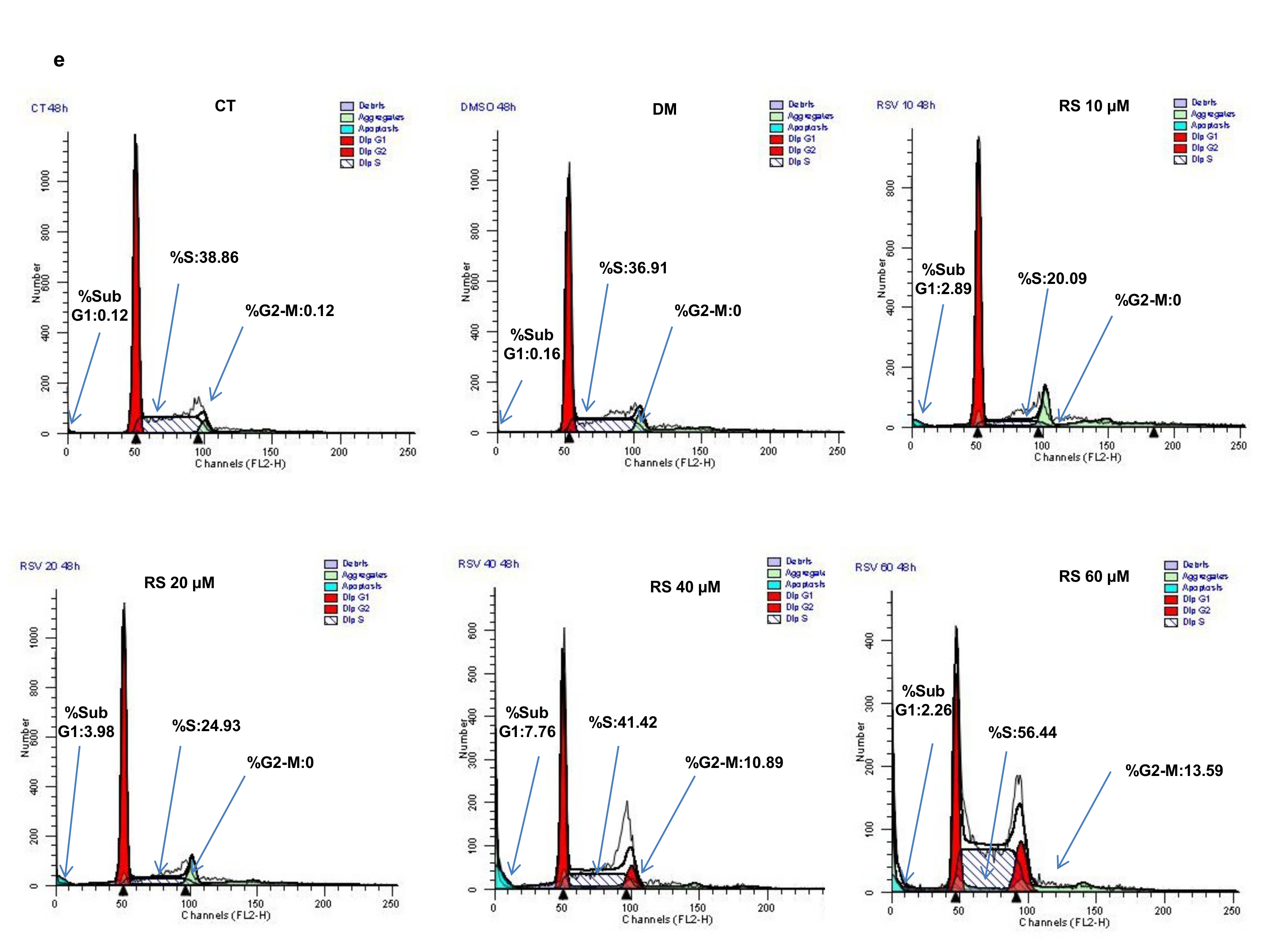
**

**Supplementary Figure 1. Resveratrol retarded C2C12 cell growth and exerted a cytotoxic effect.** (**a**) Phase contrast phase images showing the effect of 10, 20, 40 and 60 µM resveratrol (RS) on C2C12 cell morphology, captured at 72 h after treatment.

(**b**) Effect of RS on cell proliferation as reflected by cell number at 24, 48 and 72 h after treatment. (**c**) Effect of RS on cell viability expressed as % apoptotic and necrotic cells (Sub G1 cell population), determined by propidium idodide inclusion and FACS analysis. (**d**) Graph showing proportion of cells in G0+G1 phase. (**b**) Cell number was reduced with increasing dose of RS. (**c**) All doses of RS induced an increased % sub-G1 cell population. (**d**) Compared to controls, RS 10 µM (at 48 h) induced an increased proportion of cells in G0+G1 phases and reduced those in S+G2 phases, suggesting cell cycle arrest. Higher RS concentrations (40-60 µM) induced a decrease in the proportion of cells in the G0+G1 phases, starting as early as after 24 h treatment.

Data are expressed as mean ±s.e.m. of biological triplicates. *P*-value calculated using a two-tailed Student’s *t*-test. In **b**, *: *P*<0.01 vs. CT basal (t=0); ‡: *P*<0.05 vs CT at 24, ¤: *P*<0.05 vs CT at 48h; and #: *P*<0.05 vs CT 72h. In **c,** *: *P*<0.05 vs CT. In **d**, *: *P*<0.05 vs CT; **: *P*<0.01 vs CT; ***: *P*<0.005 vs CT. DMSO does not differ significantly from CT. Bars 20 *µ*m~~.~~

*Supplementary Figure 2*

**
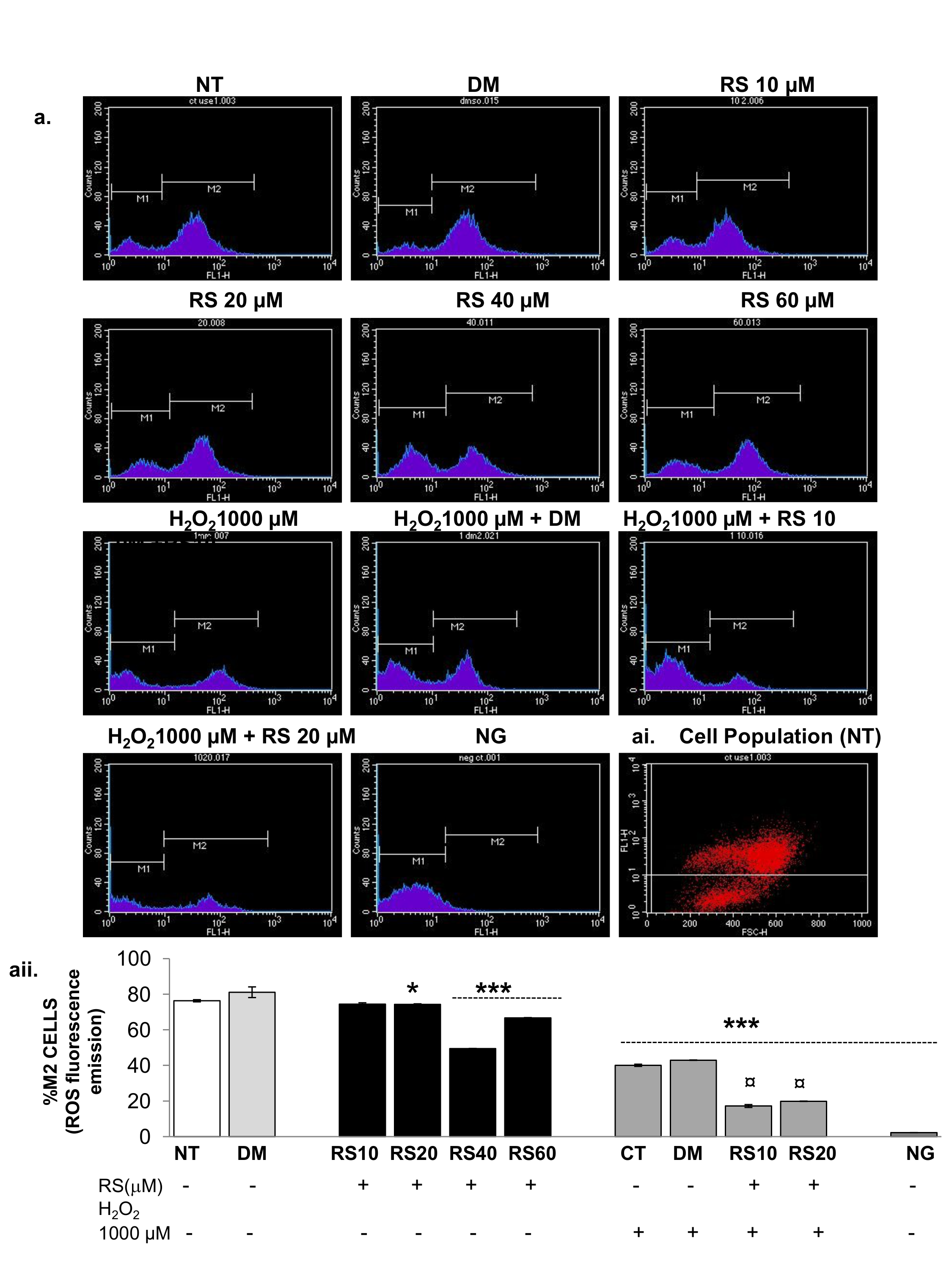
**

**Supplementary Figure 2. The antioxidant effect of resveratrol is dose dependent**

(**a**) Measure of intracellular ROS production following 24 h of treatment with 10, 20, 40 and 60 µM RS, or 1000 µM H_2_O_2_ compared to baseline ROS levels in controls (NT). FACS results were analyzed by "M2 and M1 percentage" of fluorescence variation. Cells without DCFH-DA treatment served as negative control (NG) to set M1 (non-stressed cells) and M2 (stressed) boundary.

(**ai**) Representative images of the M1 and M2 cell populations in control cells (NT) depicted by fluorescence of DCF on FACS-FL-1 channel (525 nm). (**aii**) Graph showing % M2, representing fluorescence emission for ROS species. RS-treated cells (20, 40 and 60 µM) showed a reduction of intracellular ROS, which was not significant at the lowest doses (10 µM). After 24 h treatment with 1000 µM H_2_O_2_, the surviving cells showed a marked reduction of intracellular ROS compared to CT only. RS pre-conditioning further reduced intracellular ROS level as a consequence of 1000 µM H_2_O_2_ even at the lowest doses (10 µM).Data are expressed as mean ± s.e.m. of biological triplicates. *P*-value calculated using a two-tailed Student’s *t*-test.*: *P*=0.03 vs untreated cells (NT); ***: *P*<0.0001 vs NT; ¤: *P*<0.01 vs 1000 µM H_2_O_2_. DMSO (DM) did not differ significantly from NT.

*Supplementary Figure 3*

**
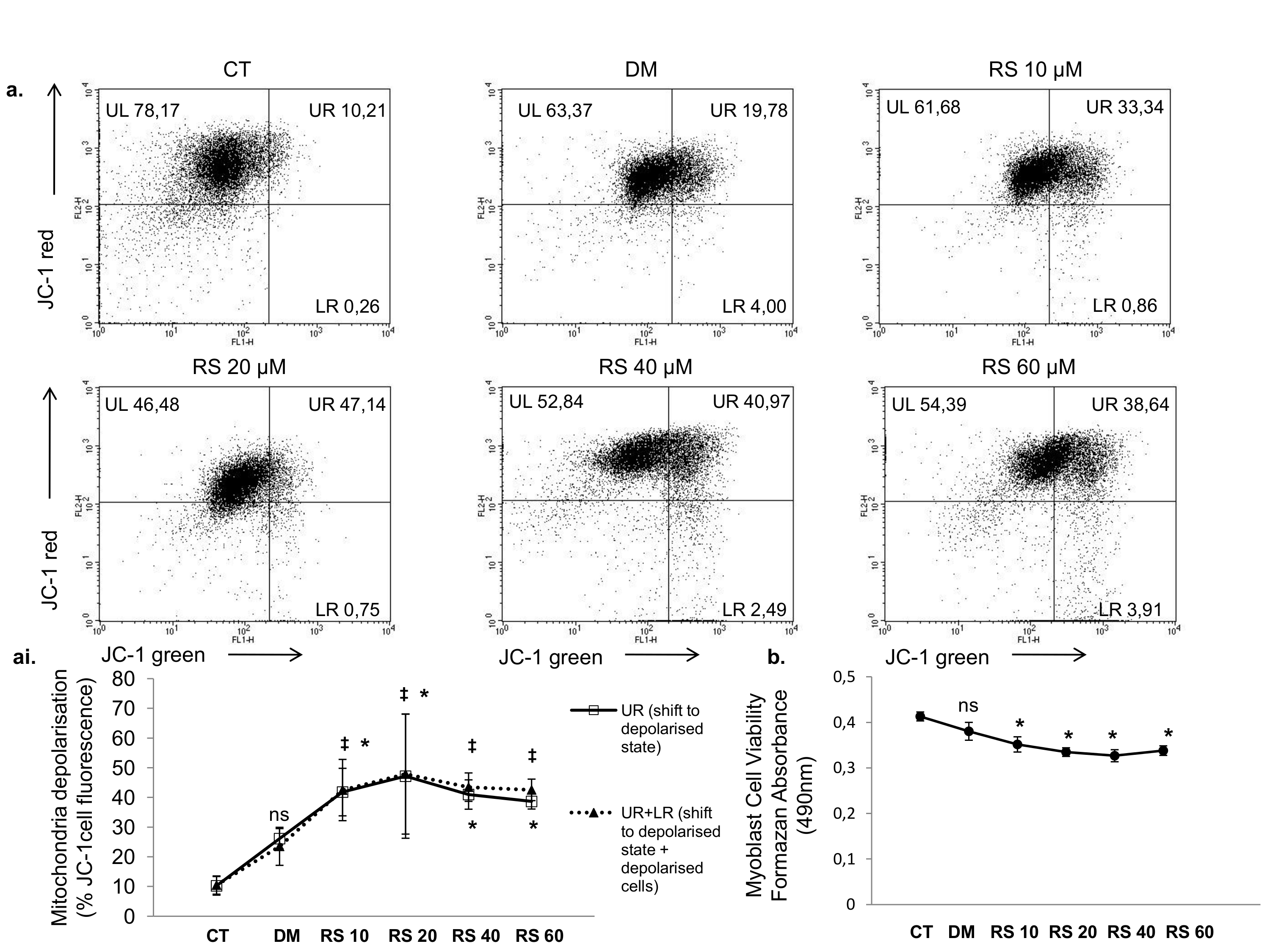
**

**Supplementary Figure 3. Resveratrol induced mitochondrial membrane depolarisation in C2C12 myoblasts**

Analysis of mitochondrial membrane depolarisation, indicated by a fluorescent emission shift from green (~525 nm) to red (~590 nm). **a**) Representative images of each condition and the cell percentages in each gate (*Upper Left*-polarised mitochondria, UL; *Upper Right*-mixed cell population i.e. polarised and depolarised mitochondria, UR; *Lower Right*-depolarised mitochondria, LR). The total % JC-1 green fluorescence cell population, including shift in depolarisation (gate UR+LR) was calculated. Percentages of depolarised cells under different conditions are reported in the graph (**ai**). (**b**) Graph showing cell viability of myoblasts after 24 h RS treatment. 24 h RS induced mitochondrial membrane depolarisation, which was higher at higher RS doses. This was associated with significant reduction of cell viability. Data are expressed as mean ±s.e.m. of biological quadruplicate. *P*-values calculated using a two-tailed Student’s *t*-test. In **ai**; gate UR+LR (shift to depolarised state+depolarised cells): ‡: *P*<0.05 vs. CT; gate UR (shift to depolarised state): *: *P*<0.04 vs CT. In **b**, *: *P*<0.05 vs CT; ns: no statistical differences vs. CT.
